# EZH2 is a prognostic factor associated with tumor stemness and immune infiltration in skull base chordoma

**DOI:** 10.1016/j.gendis.2023.101133

**Published:** 2023-10-11

**Authors:** Qian Liu, Mingxuan Li, Yujia Xiong, Yutao Shen, Tianshun Ma, Tianhao Zhang, Xiaohong R. Yang, Yazhuo Zhang, Jiwei Bai

**Affiliations:** a Beijing Neurosurgical Institute, Capital Medical University, Beijing 100070, China; b Department of Neurosurgery, Beijing Tiantan Hospital, Capital Medical University, Beijing 100070, China; c Division of Cancer Epidemiology and Genetics, National Cancer Institute, NIH, DHHS, Bethesda, MD 20892, USA

Enhancer of zeste homolog 2 (EZH2), an epigenetic regulator that inhibits transcription primarily through trimethylation of Lys-27 in histone 3(H3K27me3), has been reported to play an important role in the regulation of cell cycle, autophagy, and apoptosis, and DNA damage repair.^1^ EZH2 is highly expressed in a variety of cancers including prostate cancer, breast cancer, and renal clear cell carcinoma, and is associated with adverse prognosis.^1^ Moreover, it has been reported that EZH2 might increase tumor stemness and metastasis capacity and inhibit antitumor immunity by affecting T cells, NK cells, macrophages, and immune checkpoints.^2^^,^^3^ However, the prognostic value and the mechanism of EZH2 have not been investigated in chordoma. In this study, RNA sequencing and whole genome sequencing data based on 48 skull base chordomas as well as immunohistochemistry of paraffin sections were analyzed to elucidate the role of EZH2 in skull base chordoma. We found copy number gain mediated high EZH2 expression is associated with tumor stemness, M2 macrophage infiltration, and adverse prognosis in chordoma. Moreover, we demonstrated that EZH2 promotes the proliferation, migration, and invasion of chordoma cells in *in vitro* cell experiments.

We first analyzed the RNA sequencing data of skull base chordoma that our previous study reported,^4^ and found high EZH2 expression was associated with shorter progression-free survival (*P* = 0.016; Fig. 1A). To explore the mechanism of EZH2 in chordoma, we further analyzed the differentially expressed genes (*P* < 0.05, |log fold change (FC)| > 0.5) between the high and low EZH2 groups and found significant differences in the expression of histone coding genes between the two groups (Fig. S1A). Kyoto Encyclopedia of Genes and Genomes (KEGG) and gene set enrichment analysis (GSEA) analysis based on differentially expressed genes were also performed and the results revealed that EZH2 was correlated with several stemness-associated pathways, including signal pathway regulating pluripotency of stem cells pathway, cell cycle pathway, E2F targets, MYC targets, and G2M checkpoints pathway (Fig. 1B; Fig. S1B–D). Moreover, the stemness score calculated by single-sample gene set enrichment analysis (ssGSEA) was significantly higher in the high EZH2 group (Fig. 1C; *P* = 0.039). Besides, EZH2 expression was positively correlated with the stemness score (called mRNAsi) calculated by the one-class logistic regression algorithm (Fig. 1D; *P* = 0.017, *R* = 0.34). We also found that EZH2 was significantly positively correlated with several stemness markers, such as CXCR4, MYC, CD44, and ICAM1 (Fig. S1E–H; *P* < 0.05, *R* > 0.3). Taken together, these results demonstrated that EZH2 is strongly correlated with tumor stemness in skull base chordoma.

**Figure 1:**
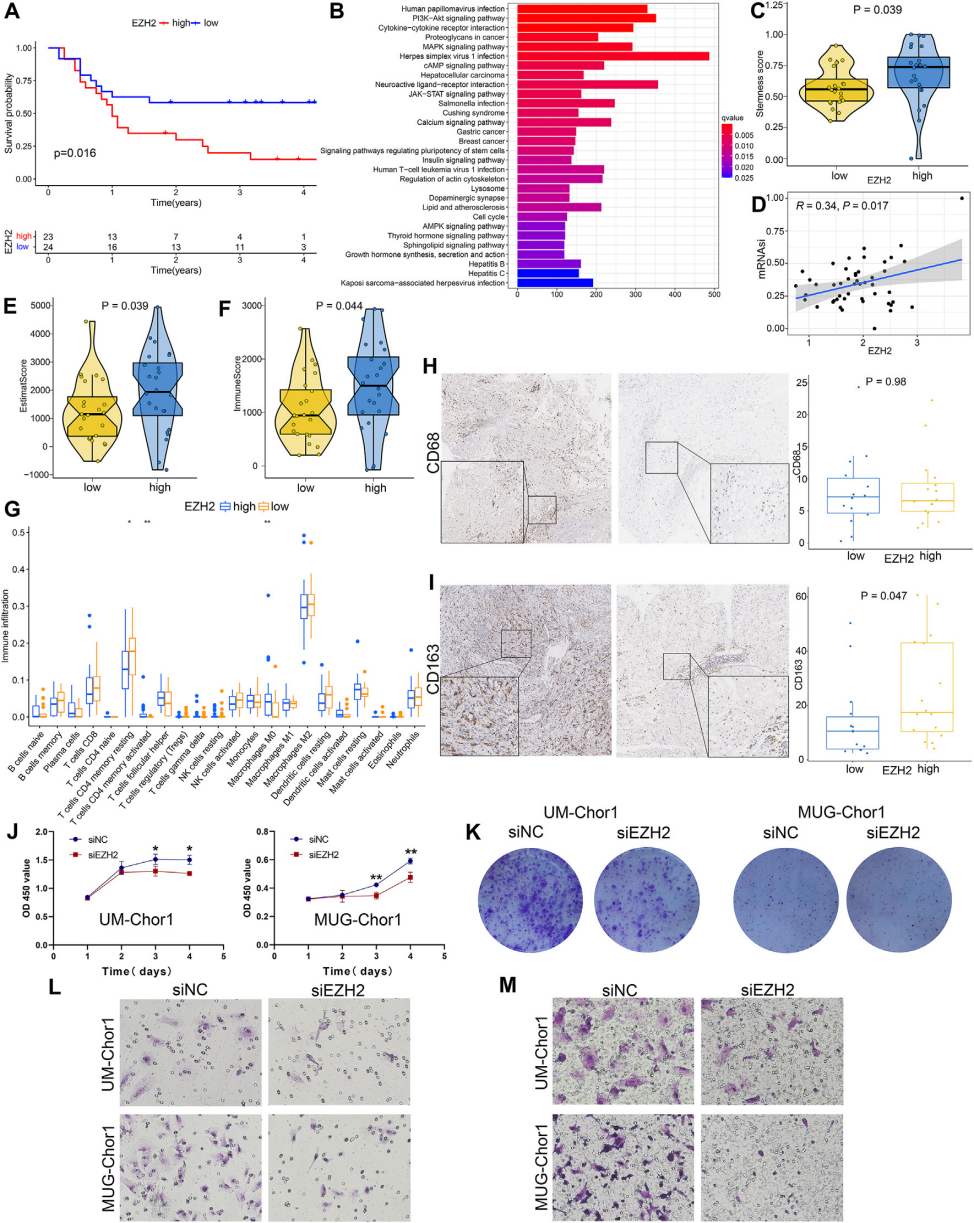
EZH2 is associated with tumor stemness and M2 macrophage infiltration and promotes tumor progression in skull base chordoma. **(A)** Kaplan–Meier analysis showed patients with high EZH2 expression had a shorter progression-free survival. **(B)** KEGG enrichment analysis of significantly different genes between the high and low EZH2 groups. **(C)** The stemness scores calculated by single-sample gene set enrichment analysis (ssGSEA) were significantly different between the high and low EZH2 groups. **(D)** EZH2 expression was positively correlated with the stemness score calculated by the one-class logistic regression (OCLR) algorithm. **(E, F)** The ESTIMATE score and immune score between the high and low EZH2 groups. **(G)** The distribution of 22 immune cell scores calculated by Cibersort between the high and low EZH2 groups. **(H, I)** Immunohistochemical staining of CD68 and CD163 in skull base chordoma tissues revealed increased CD163-positive M2 macrophages in the high EZH2 group. Left: Representative image of dense positive cells. Middle: Representative image with few positive cells. **(J)** CCK8 results of UM-chor1cells and MUG-Chor1 cells with or without EZH2 knockdown. **(K)** The number of cell colonies significantly decreased after the EZH2 knockdown in UM-chor1cells and MUG-Chor1 cells. **(L)** The migration of UM-Chor1 and MUG-Chor1 cells with or without EZH2 knockdown. **(M)** The invasion of UM-Chor1 and MUG-Chor1 cells with or without EZH2 knockdown. ^∗^*P* < 0.05, ^∗∗^*P* < 0.01.

Next, we focused on the association between EZH2 and tumor immune microenvironment in chordoma. We performed the ESTIMATE analysis to evaluate tumor immune infiltration and tumor purity, and the result showed the immune score and ESTIMATE score were significantly higher in patients with high EZH2 (Fig. 1E, F). Meanwhile, Cibersort and xCell were used to estimate the amount of each type of immune cell, and both two methods found that macrophages were more abundant in the high EZH2 group (Fig. 1G; Fig. S2A; *P* < 0.05). Other immune cells, such as B cells and T cells, were also found to be different between the high and low EZH2 groups, but the results of the two methods were inconsistent (Fig. S2A). We then performed immunohistochemical staining on available paraffin-embedded samples of RNA sequencing patients to verify the difference in macrophages. The results showed no significant difference in CD68-positive M1 macrophages (Fig. 1H), while CD163-positive M2 macrophages were significantly increased in the high EZH2 group (Fig. 1I; *P* = 0.047). In addition, EZH2 is positively correlated with several polarization factors and markers of M2 macrophage (Fig. S2B). Moreover, several inflammatory and immune-related pathways, such as inflammatory response, JAK-STAT signaling, TNFα signaling via NFKB, and PI3K-Akt signaling were also enriched in the high EZH2 group (Fig. 1B; Fig. S3A–C). We further analyzed the correlation between EZH2 and key genes of two pathways (JAK-STAT and PI3K-Akt pathway) and the results showed that EZH2 was positively correlated with JAK2, JAK3, STAT1, and STAT2 (Fig. S3D). In addition, EZH2 was positively correlated with multiple immune checkpoints (Fig. S3E). Given that tumor mutation burden was closely related to the response to immunotherapy, we also compared tumor mutation burden between the two EZH2 groups, though no significant difference between the two groups was found (Fig. S3F). Collectively, our data revealed that EZH2 affected immune infiltration; specifically, high EZH2 expression may result in more M2 macrophage infiltration in skull base chordoma.

We further explored the potential mechanism leading to high EZH2 expression. Our previous study of whole genome sequencing data did not find EZH2 gene mutations in skull base chordoma patients; However, we found that the gain of chromosome 7q, where the EZH2 gene is located, was a significant arm-level somatic copy number alteration event.^5^ Therefore, we compared EZH2 expression in patients with or without gain of chromosome 7q36.1 and found that patients with gain of 7q36.1 had higher EZH2 expression (Fig. S3G), suggesting copy number gain may contribute to the high EZH2 expression in skull base chordoma.

Lastly, we validated the function of EZH2 in two chordoma cell lines (UM-Chor1 and MUG-Chor1). Cell counting kit-8 assay revealed that the proliferation ability of chordoma cells significantly decreased after the knockdown of EZH2 using siRNA in chordoma cells (Fig. 1J; Fig. S4). Similarly, reduced cell colonies were observed in the EZH2 knockdown group compared with the control group (Fig. 1K). Moreover, the transwell assay demonstrated that the invasion and migration of chordoma cells in the EZH2 knockdown group were visibly reduced compared with the cells in the control group (Fig. 1L, M).

In summary, our study revealed the prognostic value of EZH2 in skull base chordoma and found that EZH2 is correlated with tumor stemness and M2 macrophage infiltration, providing a theoretical basis for the application of EZH2 inhibitors in skull base chordoma.

## Ethics declaration

This study was approved by the Ethics Committee of the Beijing Tiantan Hospital, Capital Medical University, Beijing, China.

## Conflict of interests

The authors declare no conflict of interest.

## Funding

This study was supported by the National 10.13039/501100001809Natural Science Foundation of China (No. 82272939, 82071559), the Capital's Funds for Health Improvement and Research (China) (2022-2Z-1076), and Beijing Hospitals Authority Youth Programme of China (QML20200502).

## Data availability

The whole genome sequencing and RNA sequencing data analyzed in this study have been deposited in the Database of Genotypes and Phenotypes (dbGaP) under Accession Code phs002301.v1.p1.

## Figures and Tables

**Figure S1:**
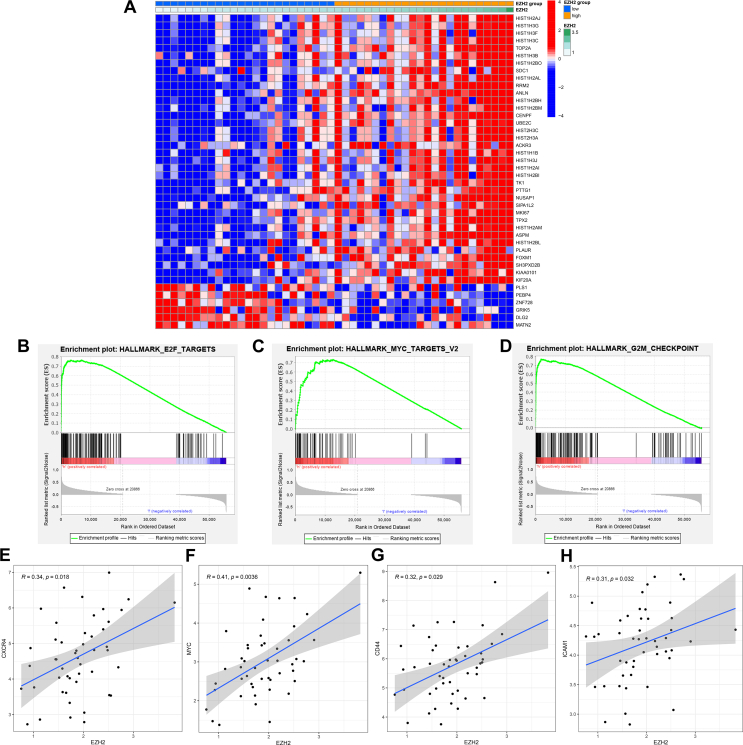
EZH2 is correlated with tumor stemness in skull base chordoma. **(A)** Heatmap of differentially expressed genes between the high and low EZH2 groups. **(B**–**D)** Gene set enrichment analysis (GSEA) revealed several stemness-associated pathways were enriched in the high EZH2 group. **(E**–**H)** EZH2 was significantly positively correlated with several stemness markers (CXCR4, MYC, CD44, and ICAM1).

**Figure S2:**
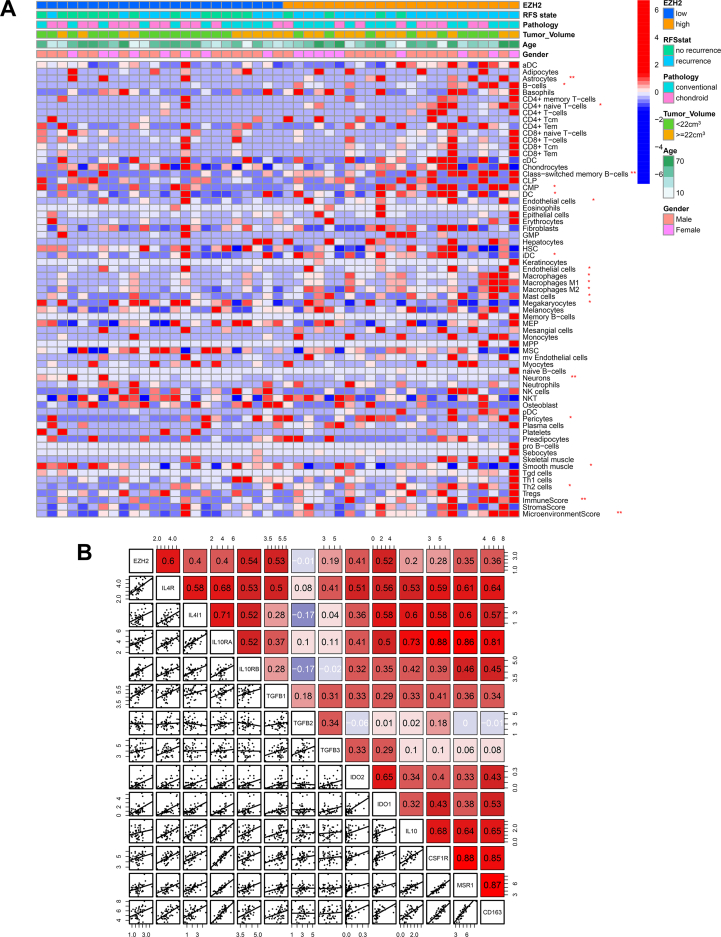
EZH2 is associated with immune infiltration in skull base chordoma. **(A)** Heatmap showed the differences in immune cell and matrix composition calculated by xCell between the high and low EZH2 groups. **(B)** The correlation between EZH2 and polarization factors and markers of M2 macrophages. ∗*P* < 0.05, ∗∗*P* < 0.01.

**Figure S3:**
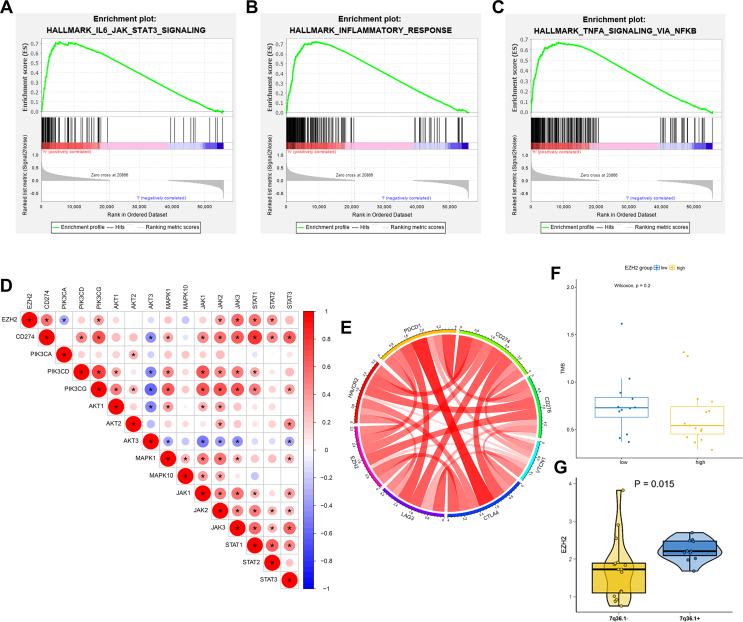
Copy number gain mediated high EZH2 expression is positively correlated with multiple immune checkpoints. **(A**–**C)** GSEA pathway analysis stratified by EZH2 expression. **(D)** Correlation between EZH2 and hub genes of JAK-STAT and PI3K-AKT pathways. **(E)** Correlation between EZH2 and immune checkpoints. **(F)** Tumor mutation burden between the high and low EZH2 groups. **(G)** Differences in EZH2 expression in patients with or without chromosome 7q36.1 amplification.

**Figure S4:**
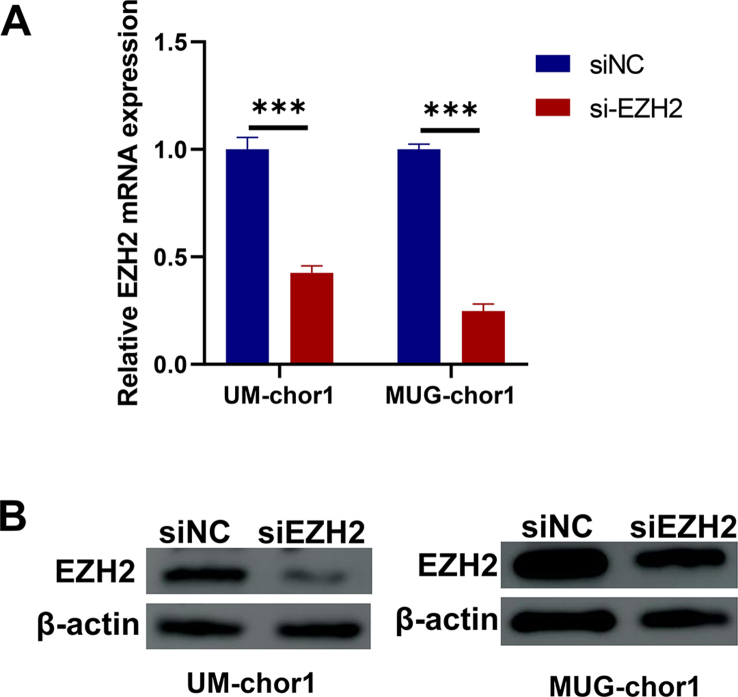
EZH2 was successfully knocked down by siRNA in chordoma cells. **(A, B)** The qRT-PCR and Western blot of EZH2 expression in UM-Chor1 and MUG-Chor1 cells with or without EZH2 knockdown. ∗∗∗*P* < 0.001.
