# Supplementary material for: EZH2 is a prognostic factor associated with tumor stemness and immune infiltration in skull base chordoma
Source: Genes Dis. 2023 Oct 11;11(5):101133. doi: 10.1016/j.gendis.2023.101133 (PMC11176624; doi:10.1016/j.gendis.2023.101133)
Supplement: Multimedia component 1 [file mmc1.docx]

**Materials and Methods**

**Patient population**

48 patients diagnosed with skull base chordoma who underwent transnasal endoscopic tumor resection surgeries at Beijing Tiantan Hospital were included in this study, as previously described.^1^ RNA sequencing data and WGS data were acquired as previously reported.^2^ The study protocols were approved by the ethics committee of the Beijing Tiantan Hospital, Capital Medical University.

**Calculation of the stemness score**

The one-class logistic regression (OCLR) and single-sample gene set enrichment analysis (ssGSEA) were used to calculate the stemness score. The OCLR stemness signature was generated by the gelnet (version 1.2.1) package in R 3.6.3.^3^ Then, Spearman correlations between mRNA expressions and the weight vectors of the stemness signature were calculated. After transformation calculation, the OCLR index was mapped to the range of 0 to 1. The ssGSEA score was estimated using the GSVA package in R.^4^ After normalization, the ssGSEA score was also mapped to the range of 0 to 1.

**Differential analysis and functional enrichment analysis**

Limma R package was used to perform differentially expressed gene (DEG) analysis based on RNA-seq data.^5^ Genes with p<0.05 and |log fold change (FC)|>0.5 were considered DEGs. Then, we used the clusterProfiler R package to perform Kyoto Encyclopedia of Genes and Genomes (KEGG) pathway analyses of DEGs.^6, 7^ In addition, GSEA was also used to analyze KEGG and Hallmark pathways between high stemness score and low stemness score groups.^8^ P < 0.05 was considered significant.

**Estimation of tumor immune microenvironment**

ESTIMATE, Cibersort, and xCell algorithms were used to evaluate the stromal and immune cell infiltration in each chordoma sample. Specifically, the Estimate R package was used to calculate the stromal score, immune score, and ESTIMATE score. We also calculated the proportions of 22 types of immune cells using CIBERSORT and leucocyte signature matrix 22 (LM22).^9^ To further obtain the levels of other stromal cells in chordoma, we analyzed the expression matrix with the xCell algorithm.^10^ Then, we compared the proportion of immune cells between the high EZH2 and low EZH2 groups.

**Immunohistochemical staining**

Tumor tissue was cut into 5μm thick sections from formalin-fixed and paraffin-embedded tissue samples of 30 chordoma patients with RNA-seq data available. Immunohistochemical staining was performed using Leica BOND II automated system with primary antibody (anti-CD68 antibody, ab283316, Abcam, 1:10000; anti-CD163 antibody, 93498S, Cell Signaling Technology, 1:200) and Bond Polymer Refine Detection (Leica Biosystems, DS9800) secondary antibody. Leica Aperio AT2 scanner was used to scan Immunohistochemical images at 400× magnification. Leica Aperio ImageScope software (version 12.3) was used to evaluate the percentage of positive cells (positive staining cells/all cells×100%).

**Cell Culture,** **Cell Counting Kit-8 (CCK-8), Cell Migration and Invasion,** **Colony Formation Assays**

MUG-Chor1 and UM-Chor1 chordoma cell lines were cultured in IMDM /1640-RPMI (4:1, Gibco) with 10% fetal bovine serum as our previous study reported.^11^ The small interfering RNAs (siRNAs) (RiboBio Medical Biotechnology) were used for EZH2 knockdown.

For the Cell Counting Kit-8 (CCK-8) assay, MUG-Chor1 cells (4×10^3^ cells/well) and UM-Chor1 cells (2.5×10^3^ cells/well) with transfection of EZH2 siRNA (siEZH2) or control siRNA (siNC) were plated in the 96-well plates, 10 μl CCK-8 was added to each well at several time points (1,2,3,4 days) and the optical density value at 450 nm was measured after 2 hour incubation.

After transfection, MUG-Chor1 cells (5×10^4^ cells/well) and UM-Chor1 cells (2.5×10^4^ cells/well) were seeded in the Transwell chamber (Corning) with or without Matrigel for cell migration and invasion assay. After 48 hours, the cells at the lower surface were fixed with paraformaldehyde and stained with crystal violet.

For colony formation assays, 2×10^3^ chordoma cells transfected with siEZH2 or siNC were plated in a 6-well plate and incubated for 14 days. The colonies were fixed with paraformaldehyde and stained with crystal violet.

**Quantitative Reverse Transcription Polymerase Chain Reaction(****qRT-PCR) and Western Blot**

Proteins and RNA were extracted after transfection of siRNA for 2 days. For qRT-PCR, we used the SuperScript III First-Strand Synthesis System to synthesize cDNA. QuantStudio 5 was used to perform qRT-PCR. Then, we normalized the expression of EZH2 according to GAPDH. The primers were as follows: GAPDH: 5’-GGAGCGAGATCCCTCCAAAAT-3’ (Forward) and 5’-GGCTGTTGTCATACTTCTCATGG-3’(Reverse), EZH2: 5’-AATCAGAGTACATGCGACTGAGA -3’ (Forward) and 5’- GCTGTATCCTTCGCTGTTTCC-3’ (Reverse). 10% sodium dodecyl sulfate-polyacrylamide gel electrophoresis was used to separate protein samples. Then, we transferred protein blots to polyvinylidene difluoride membranes and incubated the bands with the primary antibodies (anti-EZH2, 5246S, Cell Signaling Technology, 1:1000) overnight and the following secondary antibodies for 1 hour. Amersham Imager 600 was used for chemiluminescence.

**Statistical analysis**

All statistical analyses were performed with R software (version 3.6.3). Kaplan-Meier analysis and univariate Cox analysis were implemented with the Survival and survminer R package. The Wilcox test was used to compare the difference in stemness scores across different clinical characteristics. Spearman's rank correlation was used to test for correlation between EZH2 and other genes (p<0.05 and cor>0.3 was considered significantly correlated). And correlation matrixes were plotted using ggplot2, circlize, and corrplot R packages. p <0.05 was considered significant in all comparisons.

**References**

1 Bai J, Shi J, Zhang Y, et al. Gene Expression Profiling Identifies Two Chordoma Subtypes Associated with Distinct Molecular Mechanisms and Clinical Outcomes [J]. Clin Cancer Res, 2023, 29(1): 261-270.

2 Bai J, Shi J, Li C, et al. Whole genome sequencing of skull-base chordoma reveals genomic alterations associated with recurrence and chordoma-specific survival [J]. Nat Commun, 2021, 12(1): 757.

3 Malta TM, Sokolov A, Gentles AJ, et al. Machine Learning Identifies Stemness Features Associated with Oncogenic Dedifferentiation [J]. Cell, 2018, 173(2): 338-354.e315.

4 Miranda A, Hamilton PT, Zhang AW, et al. Cancer stemness, intratumoral heterogeneity, and immune response across cancers [J]. Proc Natl Acad Sci U S A, 2019, 116(18): 9020-9029.

5 Smyth GK, Michaud J, Scott HS. Use of within-array replicate spots for assessing differential expression in microarray experiments [J]. Bioinformatics (Oxford, England), 2005, 21(9): 2067-2075.

6 Kanehisa M, Furumichi M, Tanabe M, et al. KEGG: new perspectives on genomes, pathways, diseases and drugs [J]. Nucleic Acids Res, 2017, 45(D1): D353-d361.

7 Wilkerson MD, Hayes DN. ConsensusClusterPlus: a class discovery tool with confidence assessments and item tracking [J]. Bioinformatics (Oxford, England), 2010, 26(12): 1572-1573.

8 Subramanian A, Tamayo P, Mootha VK, et al. Gene set enrichment analysis: a knowledge-based approach for interpreting genome-wide expression profiles [J]. Proc Natl Acad Sci U S A, 2005, 102(43): 15545-15550.

9 Newman AM, Liu CL, Green MR, et al. Robust enumeration of cell subsets from tissue expression profiles [J]. Nature methods, 2015, 12(5): 453-457.

10 Aran D, Hu Z, Butte AJ. xCell: digitally portraying the tissue cellular heterogeneity landscape [J]. Genome biology, 2017, 18(1): 220.

11 Xiong Y, Li M, Shen Y, et al. PALB2 as a factor to predict the prognosis of patients with skull base chordoma [J]. Front Oncol, 2022, 12: 996892.
